# Pulmonary Rehabilitation for COPD

**Published:** 2017

**Authors:** Nicholas Hopkinson

**Affiliations:** NIHR Respiratory Biomedical Research Unit at Royal Brompton and Hare field HNS Foundation Trust and Imperial College London

Given finite healthcare resources it is important to ensure that they are put to the best use. Value in health care represents the relationship between health outcomes achieved and resources used. In a highly prevalent condition it is important to ensure that the highest value interventions are employed effectively In COPD these are influenza vaccination, smoking cessation and pulmonary rehabilitation (www.impressresp.com).

Skeletal muscle impairment is a common and important feature of respiratory disease. In COPD it is associated with reduced quality of life, exercise capacity and survival. Muscle endurance is also reduced and this has been confirmed using non-volitional techniques (magnetic femoral nerve stimulation). Muscle fatigue is an important symptom limiting exercise. The main driver is physical inactivity and this occurs in early in the course of the disease in particular this may be before it has been diagnosed. Physical inactivity may itself drive lung disease progression.

As well as a loss of muscle bulk there is a shift away from a Type I fibre, oxidative endurance muscle phenotype. The underlying biology is complex and in addition to inactivity, inflammation, corticosteroids, reduced anabolic hormones, corticosteroid treatment, hypoxia, poor nutrition and increased resting energy expenditure may all play a role. There is a recent ATS/ERS statement on limb muscle dysfunction in COPD.

Exercise has a wide range of beneficial effects. It can improve exercise capacity, lipid profile, reduce falls, reduce cardiac risk, improve depression, insulin sensitivity, and systemic inflammation as well as protecting against cognitive decline and osteoporosis.

Pulmonary rehabilitation has been defined as “an evidence-based, multidisciplinary, and comprehensive intervention for patients with chronic respiratory diseases who are symptomatic and often have decreased daily life activities. Integrated into the individualized treatment of the patient, pulmonary rehabilitation is designed to reduce symptoms, optimize functional status, increase participation, and reduce health care costs through stabilizing or reversing systemic manifestations of the disease.”

A standard PR course might be ∼8 weeks of twice weekly sessions as well as home exercise being encouraged. A mixture of aerobic and strength training is used and it is supervised by health professionals. The venue can be a hospital gym or a community gym or other space. It should be integrated into clinical management and include education about self-management and other issues.

There is a compelling evidence base for benefit of pulmonary rehabilitation in COPD, reversing the effects of deconditioning improving exercise capacity and quality of life. The cost per QALY of pulmonary rehabilitation is significantly better than for pharmacotherapy in COPD.

Pulmonary rehabilitation in the early post-exacerbation phase reduces readmission and improves survival with a number needed to treat of 4 to prevent one readmission.

This recent review on PR for non-COPD lung disease is useful.

One way to improve pulmonary rehabilitation uptake is the use of a COPD discharge care bundle has been developed with input from guidelines, clinicians and patients which includes the following items:

- Smoking cessation
- Inhaler technique
- Written self-management plan
- Assessment for pulmonary rehabilitation
- Follow up arrangement made before discharge

Patients also complete a safe discharge checklist with the nurse responsible for their care. Evidence suggests that it may reduce readmission rates and improve quality of care. The documents are all available in the online supplement (open access).

